# Direct Measurement of Transverse Compressive Properties of Polyacrylonitrile-Based Single Carbon Fibers

**DOI:** 10.3390/ma18133018

**Published:** 2025-06-25

**Authors:** Jin Yan, Hongyi Ma, Xueming Wang, Hongyun Li, Biao Li, Qi Wei, Zhenghua Cao

**Affiliations:** 1 Composite Testing Technology Center, AVIC Composite Co., Ltd., Beijing 101300, China; mahongyi0523@163.com (H.M.); acc_lihy@163.com (H.L.); 2 School of Aeronautics, Northwestern Polytechnical University, Xi’an 710072, China; libiao@nwpu.edu.cn; 3 Aviation Key Laboratory of Science and Technology on Advanced Surface Engineering, AVIC Manufacturing Technology Institute, Beijing 100024, China; wqweiqi163@163.com; 4 AVIC Composite Technology Center, AVIC Manufacturing Technology Institute, Beijing 101300, China; czhcaozhenghua@163.com

**Keywords:** carbon fiber, mechanical properties, transverse compression, anisotropy

## Abstract

Evaluating the properties of single carbon fibers is crucial, as it provides parameters not only for optimizing carbon fiber production processes but also for enhancing composite material properties. In recent years, significant advancement have been made in the evaluations of the transverse compressive properties of single fibers. However, compressive testing methods for fibers in the fine size range remain limited at the commercial scale. The direct measurement of the transverse compressive properties of three domestic high-strength polyacrylonitrile-based single carbon fibers (T300 grade, T700 grade, and T800 grade) with diameters of ~5.5~6.5 μm was made possible through the use of a micro-compression tester. Transverse compressive moduli of 5.19 GPa (0.050), 5.42 GPa (0.104), and 6.63 GPa (0.120) were obtained for domestic T300-, T700- and T800-grade carbon fibers, respectively. In addition, transverse compressive strengths of 2.35 GPa (0.033), 2.65 GPa (0.041), and 2.82 GPa (0.121) were obtained for domestic T300-, T700- and T800-grade carbon fibers, respectively. It is noted that minor deviations in fiber geometry from an ideal circular cross-section may influence absolute modulus and strength values. These fibers exhibit strong anisotropy in the longitudinal and transverse directions from the fiber axis. A correlation between the transverse compressive modulus and strength was found for these fibers. These results provide critical parameters for the optimization of carbon fiber-reinforced composite designs (e.g., enhancing impact resistance in aerospace structures), offering substantial practical value to the field of materials science.

## 1. Introduction

The increasing application of carbon fiber-reinforced polymer (CFRP) composites in aircraft structures signifies that low-cost composite technologies have entered a new phase of engineering applications [1,2,3,4]. Currently, high-performance composite materials are utilized in the aviation industry. However, there has been little improvement in the compressive performance of CFRP composites over the past 30 years [5,6]. The compressive strength of carbon fibers and their polymer composites remains at only 30–50% of their tensile strength due to their strong anisotropy property [7,8]. The low compressive strength/modulus, particularly in the transverse direction, severely limits their practical applications [9,10,11].

Furthermore, with the accumulation of experience in composite material applications, damage resistance to low-energy impacts has become a primary design consideration for aircraft composite structures [12,13]. During the development phase of a unidirectional tape composite structure, the damage resistance performances severely affected both the safety and cost-effectiveness of the composite material’s applications [14,15]. Based on previous research, fiber fracture, particularly axial and transverse compressive failure, constitutes one of the primary fracture modes in low-velocity impact damage events of CFRP laminates [16,17]. Despite extensive research on the measurement of the transverse compressive properties of single carbon fibers, critical gaps persist. Existing micro-compression techniques for single carbon fibers face limitations in accuracy, standardization and reproducibility. For instance, Ward et al. [18] pioneered the used of two parallel glass plates and lever systems to measure the transverse modulus of polyethylene and single polymeric fibers with a larger diameter. The contact width under varying load levels during compression was monitored using interference fringe patterns observed under a microscope. By measuring the contact width, Poisson’s ratio and tensile modulus during compression, the transverse compressive modulus of the fiber could be estimated through calculations. However, this microscope method cannot be applied to fine-diameter fibers. Subsequent studies by Kawabata et al. [19], Cheng et al. [20] and Lim et al. [21] advanced methodologies through custom-built devices, yet these approaches lacked standardization, leading to variability in results due to inconsistent load application and machine compliance issues at low-force regimes. Thus, there remain limitations in the accurate direct measurement of the transverse compressive properties of single carbon fibers with a diameter of 5 μm to 10 μm. Naito et al. [22] established foundational correlations between microstructure and transverse properties across diverse fiber types, including PAN-based (e.g., T1000 GB, M60 JB) and pitch-based (e.g., K13D, XN-05) carbon fibers. Moreover, beyond precursor types, fiber properties are strongly influenced by processing conditions (e.g., stabilization parameters significantly modify crystallite formation [23]) and surface treatments (e.g., oxidation/sizing synergistically improves interfacial adhesion [24]). Recent advances in coupling multiscale modeling with experiments have further accelerate the cost-effective optimization of these parameters [25]. This study focuses on optimizing domestic high-strength PAN-based fibers for aerospace applications. Our methodology advances testing precision and links $E_{T}$ to *E*, and $\sigma_{T}$ to σ. For example, T800-grade fibers ($E_{T}$ = 6.63 GPa, *E* = 295 GPa, $\sigma_{T}$ = 2.82 GPa and σ = 5.6 GPa) exhibit a $E_{T}$/*E* ratio of ~2.2% and a $\sigma_{T}$/σ ratio of ~50.5%, aligning with their highly oriented graphitic structure. This correlation provides a practical framework for predicting transverse performance from tensile data, offering direct parameters for CFRP laminate design. Critically, compared to nanoindentation, direct measurement is essential for composite design validation since it reproduces actual service conditions. It captures general failure mechanisms (e.g., fiber kinking and rupture) rather than localized elastic responses [26]. It also eliminates assumptions of homogeneous material behavior [9,22], and provides strength data relevant to structural failure criteria [17]. Nanoindentation, while useful for small-strain elastic properties [26], cannot characterize compressive strength or replicate the full stress state in CFRP failure modes (e.g., delamination onset during impact [16]). Furthermore, commercial micro-compression testing machines are mainly used for measuring the compressive strength of spherical individual spherical particles [27,28,29]. While several reported studies on fiber compression testing applications have been found, there remains a need for scalable and repeatable protocols for characterizing the transverse compressive properties of single carbon fibers.

In this work, a commercial micro-compression tester was employed to address these limitations, integrating high-resolution displacement measurement (0.0001 μm), real-time optical monitoring, and standardized load application. This system is different from the prior measurement devices as it ensures precise alignment of single carbon fibers and minimizes machine compliance errors. This methodology eliminates the indirect assumptions (e.g., Poisson’s ratio estimation), enhances reproducibility through automated force-displacement control, and offers compatibility with industry-standard testing practices.

The current work hypothesizes that transverse compressive modulus and strength scale with the fiber grade of PAN-based single carbon fibers (diameters 5–8 μm) reflect intrinsic structural anisotropy. Specifically, higher-grade fibers (T800) with improved graphitic crystallite alignment and reduced defects are expected to exhibit superior transverse mechanical performance compared to lower grades (T300 and T700). This hypothesis aligns with recent advancements in fiber anisotropy characterization, where advanced microscopy and computational models have linked anisotropic behavior to microstructural features such as skin-core morphology and interlayer bonding [9,19]. Understanding these relationships is critical for optimizing CFRP composites, as transverse properties directly influence failure modes (e.g., delamination, matrix cracking) under impact loading [16,17]. For instance, composites with higher transverse compressive resistance could mitigate stress concentrations at fiber–matrix interfaces, thereby improving damage tolerance and energy absorption [10,14]. However, generalizability requires caution as fibers from different processes (e.g., pitch-based) exhibit distinct microstructures and anisotropy [22], likely altering transverse trends. Additionally, diameters outside 5–8 μm may require scaled testing protocols, as geometric effects dominate contact mechanics in compression [30].

By bridging the gap between laboratory-scale testing and industrial standardization, this work not only provides reliable transverse property data for PAN-based single-carbon fibers but also establishes a predictive framework for future studies to correlate fiber anisotropy with composite performance. These insights are vital for advancing next-generation CFRP designs, particularly in aerospace applications where damage resistance to low-energy impacts and structural reliability are paramount [12,13].

## 2. Materials and Methods

### 2.1. Materials

Three commercialized types of domestic high-strength PAN-based carbon fibers (T300, T700 and T800 grades) were selected for characterization. It should also be noted that the as-received fibers contained a sizing agent. The basic information for the carbon fibers is given in Table 1.

### 2.2. Specimen Preparation

**Figure 1:**
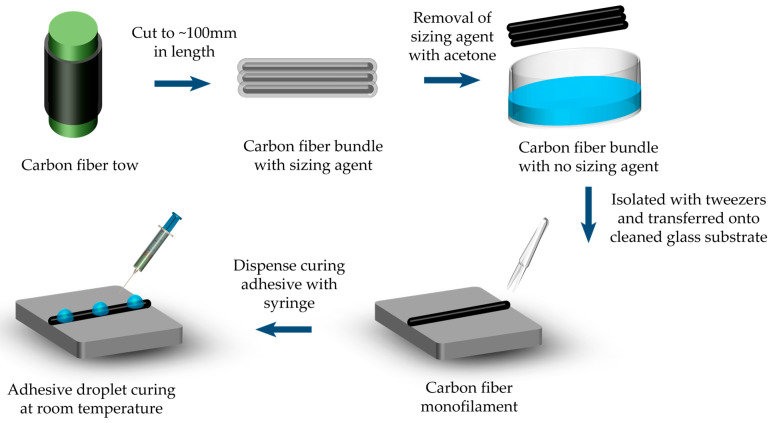
Schematic of sample preparation used for the transverse compression test of single carbon fiber.

**Figure 2:**
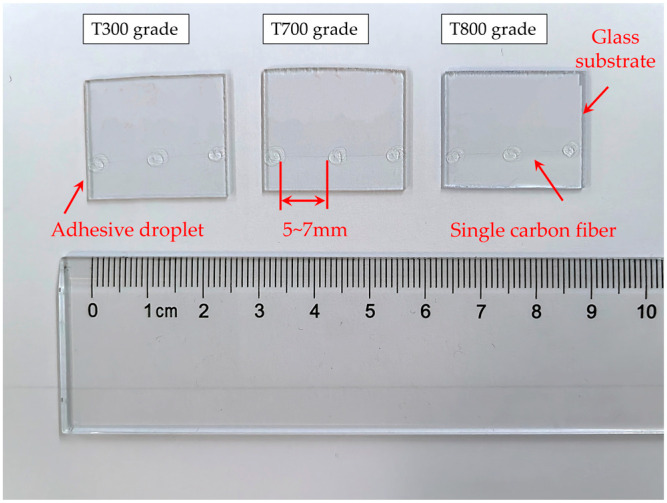
Optical images of transverse compression test samples.

The schematic of the sample preparation method used for the transverse compression test of single carbon fiber is shown in Figure 1. Typically, at least the initial three meters of fibers from each tow should be removed before sampling begins. If necessary, the surface layer of the fiber tow may also be discarded. Note that the fibers should remain undamaged during the fiber selection process, avoiding the formation of loops or knots and preserving the original twist integrity of the fibers. A carbon fiber bundle (approximately 100 mm in length) was sectioned from a continuous carbon tow. The sizing agent was removed according to ISO 10548:2002 [31] method A. Post-treatment SEM characterization confirmed complete removal, where fiber surfaces exhibited consistent topography without sizing-related residues (e.g., polymer films or agglomerates), ensuring minimal effects of interfacial artifacts on compression measurements. A single carbon fiber monofilament was isolated using anti-static tweezers and subsequently transferred onto an ultrasonically cleaned glass substrate. A room-temperature curing adhesive (Loctite, Newington, CT, USA, 401) was drawn into a syringe and subsequently dispensed as discrete droplets onto the carbon fiber monofilament, with inter-droplet spacing maintained at approximately 5–7 mm. Note that the recommended curing time for rapid room-temperature liquid curing resin is 10 to 30 minutes. Following this, the glass substrate was transferred to a clean environment to cure the adhesive droplets. Upon confirmation of complete curing, the specimen was deemed ready for transverse compression testing, see Figure 2.

### 2.3. Transverse Compression Tests

**Figure 3:**
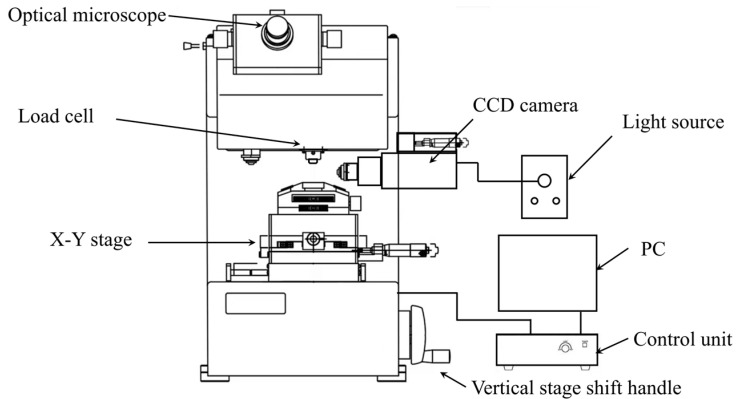
Schematic diagram of micro-compression tester system.

To measure the transverse compressive properties of polyacrylonitrile (PAN)-based single carbon fibers, the transverse compression tests were carried out on a micro-compression testing machine (Shimadzu, Kyoto, Japan, MCT-211) with a load cell range from 9.8 mN to 1961 mN. As shown in Figure 3, the bottom specimen stage and the vertical stage can be shifted along the X, Y and Z directions to align the single carbon fibers. The compressive load was induced by a 50 μm flat diamond indenter, which is capable of high-resolution displacement value measurement. The displacement ranges up to 10 μm and with 0.0001 μm of resolution. Additionally, this machine is equipped with two observation kits: the top optical microscope (OM) and the side charge-coupled device (CCD) camera. The top microscope has a magnification of 500× and can also be used for specimen dimensions (diameter and length) measurement. The side observation CCD camera was used for real-time fiber status observation during the compression tests. The high hardness (Hv > 8000) and low surface roughness (Ra < 0.01 μm) of the diamond indenter minimized local stress concentrations. Furthermore, the real-time side-view CCD monitoring confirmed full-width fiber-indenter contact prior to loading, and the Morris model intrinsically accounted for contact geometry via the half-width (Equation (4)), validated by R^2^ > 0.98 in linear regimes. Machine compliance errors were mitigated beyond initial calibration via pre-test ‘blank runs’ (without a specimen) to measure displacement offset, which was subtracted from all fiber data, and exclude discontinuous loading (>5% load drop) in the linear region, caused by system instability.

**Figure 4:**
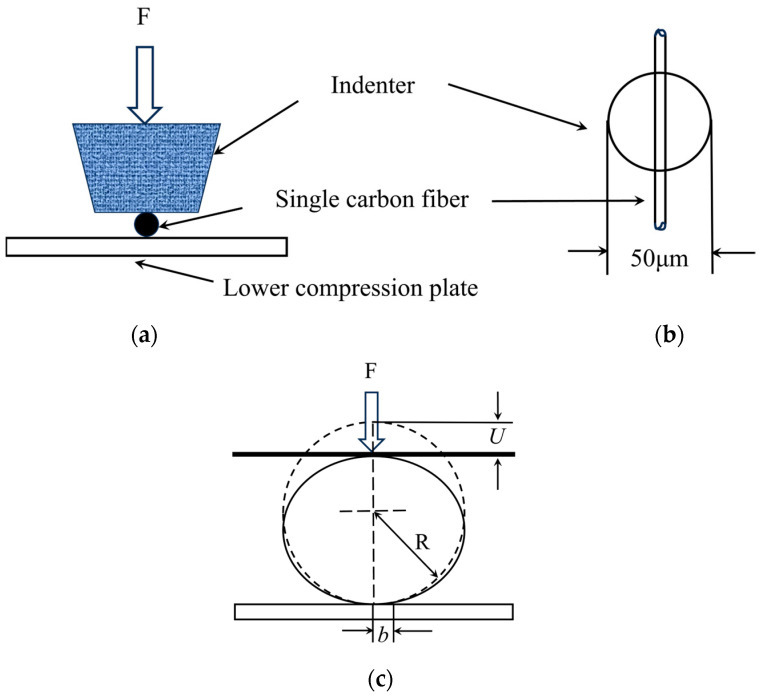
Schematic diagrams of (**a**) transverse compression test of a single carbon fiber; (**b**) indenter and single carbon fiber; and (**c**) a single carbon fiber and displacement measurements during compression.

**Figure 5:**
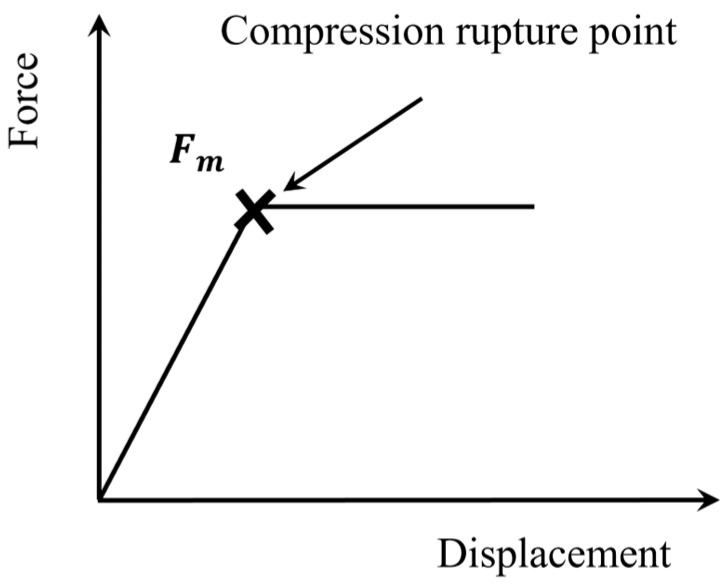
Schematic of the force–displacement curve of the single carbon fiber during compression using the micro-compression tester.

The specimen was fixed between the upper compression rod and the lower compression plate, see Figure 4. After focusing, the dimensions of the single carbon fiber were measured using the specimen dimension measurement function. Then, the indenter began to apply a compressive load on the fiber. When the indenter touched the fiber surface, the crosshead speed for the PAN-based single-carbon fiber was kept at a constant rate of 7 mN/s for all transverse compression tests [32]. When the rupture occurred, the maximum compressive force ($F_{m}$) was maintained for 5 s before the system stopped. The fracture of the fiber under loading is shown as a plateau in the force vs. displacement curve in Figure 5. The system applied force proportional to the current intensity through electromagnetic control. The deformation in the single carbon fiber was measured automatically during compression. At least 10 valid compressions at different positions were measured for each fiber batch (a minimum of 30 compressions per grade).

The relationship between displacement (U) and compression force ($F_{m}$) for a circular cross-sectional fiber was derived by Morris [30] by modeling the top load as a point source. Recent work compared various theoretical models for the transverse compressive modulus of cylindrical samples, the results show that the experimental test data was most accurately fitted by the Morris model [30]. The displacement (U) is calculated by:

(1) $$U=\frac{4F}{\pi }(\frac{1}{E_{T}}-\frac{v^{2}}{E_{L}})(\sinh^{-1}\left(\frac{R}{b}\right))$$

where F is the force per unit length for fiber, $E_{T}$ is the transverse compressive modulus of the fiber, $E_{L}$ is the longitudinal compressive modulus, v is Poisson’s ratio, R is the radius of the fiber, and b is the half-width of the contact zone, calculated as follows according to the Morris model [30]:

(2) $$b=\sqrt{\frac{4FR}{\pi }(\frac{1}{E_{T}}-\frac{v^{2}}{E_{L}})}$$

For carbon fibers, due to the anisotropic orientation in the transverse and longitudinal directions, $E_{T}$ ≪ $E_{L}$, the value of $\frac{v^{2}}{E_{L}}$ is negligible [33]. Therefore, Equations (1) and (2) can be simplified as follows for the carbon fibers:

(3) $$U=\frac{4F}{\pi }(\frac{1}{E_{T}})(\sinh^{-1}\left(\frac{R}{b}\right))$$

where

(4) $$b=\sqrt{\frac{4FR}{\pi }(\frac{1}{E_{T}})}$$

The equations can be modified by introducing the fiber diameter, *D*. Thus, the transverse compression strain $\varepsilon_{T}$ and transverse compression stress $\sigma_{T}$ can be normalized as follows [33]:

(5) $$\varepsilon_{T}=\frac{U}{D}$$

(6) $$\sigma_{T}=\frac{F}{D}=\frac{F_{m}}{l\cdot D}$$

Equations (3) and (4) can be modified as follows:

(7) $$\varepsilon =\frac{4\sigma }{\pi }(\frac{1}{E_{T}})\sinh^{-1}\left(\frac{R}{b}\right)$$

(8) $$b=\sqrt{\frac{8\sigma R^{2}}{\pi }(\frac{1}{E_{T}})}$$

Therefore, $E_{T}$ can be derived directly from the transverse compression stress–strain curve using Equations (7) and (8).

### 2.4. Characterization of Failure Mode

For real-time monitoring of the morphological changes in single carbon fibers under compressive loading, a side observation charge-coupled device (CCD) was applied during the compression process. After testing, the failure modes were characterized using a high-resolution scanning electron microscope (SEM) (Hitachi, Kyoto, Japan, SU8600) at an operating voltage of 1.0 kV.

## 3. Results and Discussions

### 3.1. Transverse Compressive Modulus

Specimens were prepared to evaluate the transverse compression properties of single carbon fibers, the dimensions (lengths and diameters) are shown in Table 1. The measured transverse compressive properties ($E_{T}$ and $\sigma_{T}$) of three domestic high-strength PAN-based carbon fibers (T300, T700 and T800 grades) are given in Table 2. The reference tensile properties (E and σ) are listed for comparison.

To ensure repeatability, three independent fiber batches (see Table 1) were tested for each carbon fiber grade (T300, T700, and T800 grades). For each batch, at least 10 valid compressions were performed at different positions along the fiber length (a minimum of 30 compressions per grade). A statistical analysis was conducted for this method. Tests exhibiting slippage, misalignment or non-contact (detected via real-time CCD monitoring) were excluded. Less than 5% of the total tests were discarded based on these criteria. The data normality was verified using the Shapiro–Wilk test, and the *p*-value was greater than 0.05 for all batches (confirming data normality). The precision and confidence intervals were analyzed. Results are reported as mean ± 95% confidence intervals (CIs) calculated using Student’s t-distribution (see Table 2). For example, T800 fibers exhibited $E_{T}$ of 6.63 GPa (95% CI is 6.51 to 6.75 GP when the sample size is 30). Moreover, the inter-batch repeatability was quantified via the coefficient of variation (CV). For $E_{T}$, CV values were 4.2% (T300), 3.8% (T700) and 4.6% (T800), thus confirming the consistency of the method.

**Figure 6:**
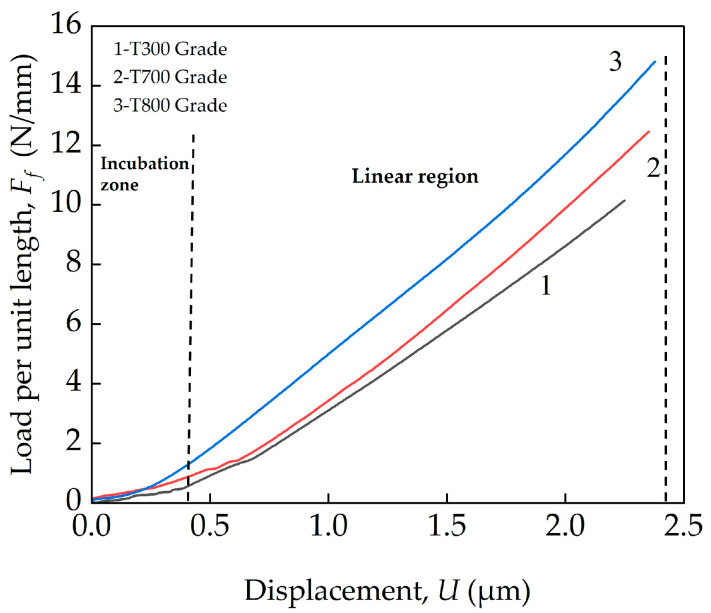
Typical load–displacement curves for three domestic PAN-based single carbon fibers (T300, T700 and T800 grades) under transverse compressive loading.

The typical transverse compression curves for three domestic high-strength PAN-based single carbon fibers (T300, T700 and T800 grades) are plotted in terms of load versus displacement in Figure 6. The transverse compression curves for the three types of fibers exhibit similar trends. Two stages that can be seen on the curves: a slow increase in load before a steady increase in displacement. In the ‘initial region’, where the load values are below ~2 N/mm, the loads increase non-linearly with the displacements. This is due to the contact zone between the single carbon fiber and the contact surfaces. The cross-section of the single carbon fiber did not exhibit perfect circularity, and porosity- and defect-induced unevenness could be found on the surface. This led to the existence of this incubation period, and similar observations were reported. At loads higher than ~2 N/mm, the load increased linearly with displacement linearly until failure. In the second region, the three types of high-strength PAN-based single-carbon fibers all exhibited linear elastic deformation. Similar behavior was also reported by Guo et al. [34], resulting from the inherent elastic deformation of the carbon fiber during the initial loading stages.

**Figure 7:**
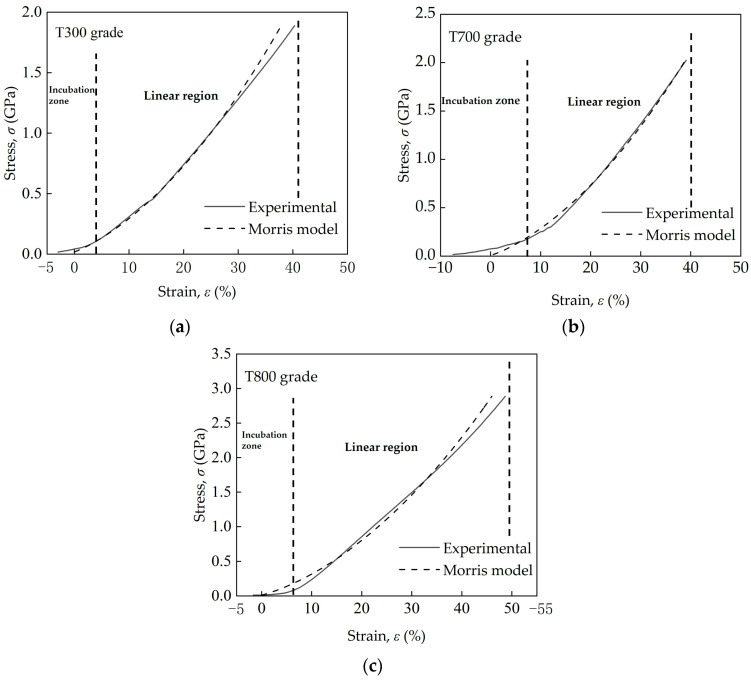
Typical stress–strain curves for three domestic PAN-based single carbon fibers–(**a**) T300, (**b**) T700 and (**c**) T800 grade–under transverse compressive loading, fitted using the Morris model.

To estimate the transverse compressive modulus, $E_{T}$, for single carbon fiber, typical stress versus strain curves were fitted using the Morris model (Equation 3), see Figure 7. The solid lines show the curve from the transverse compression tests, and the dotted lines illustrate the theoretical $\sigma_{T}$ value obtained from Equation (1). The applicability of the Morris model’s was validated by comparing the experimental stress–strain curves to theoretical fits (see Figure 7). While Equations (3)–(8) assume circular cross-sections, empirical validation confirmed the Morris model’s applicability despite minor geometrical deviations. Correction factors for contact width (Equation 4) accommodated irregularities, achieving R^2^ > 0.98 in linear regimes, indicating a good correlation between the experimental data and the Morris model. A good agreement in the linear region can be seen for the curve in the linear elastic region until brittle fracture for the three types of PAN-based single-carbon fibers. The compressive moduli for the T300, T700, and T800 grades were measured to be 5.19 GPa (standard deviation: 0.050), 5.42 GPa (standard deviation: 0.104) and 6.63 GPa (standard deviation: 0.120), respectively. While the Morris model assumes an ideal circular cross-section, minor deviations (e.g., ellipticity, surface roughness) were observed via SEM. To quantify their impact, the ellipticity ratio (E_r_ = major axis/minor axis) was calculated from SEM images for each fiber grade (T300 grade: 1.25 ± 0.03; T700 grade: 1.23 ± 0.04; and T800 grade: 1.09 ± 0.02). Elliptical cross-sections increase contact stiffness, leading to underestimated $E_{T}$ values based on nominal diameter [30]. Finite-element simulations suggest $E_{T}$ underestimations of ~10% (T300 grade), ~8% (T700 grade) and ~3% (T800 grade) relative to perfectly circular fibers. Despite these geometric effects, microstructural trends (e.g., graphitic alignment in T800) remain the primary driver of differences in $E_{T}$.

The transverse compressive modulus values (5.19–6.63 GPa) align with prior compression studies on PAN-based fibers [22] but are lower than nanoindentation-derived elastic constants. For example, Shirasu et al. [26], they utilized nanoindentation combined with finite element analysis (FEA) to determine the transverse elastic constant (C_11_) of high-strength PAN-based fibers (e.g., C_11_ is 21.6 GPa for T800SC). This discrepancy highlights the distinction between direct compressive failure mechanisms and small-strain elastic responses, underscoring the relevance of our methodology for composite applications under transverse loading.

**Figure 8:**
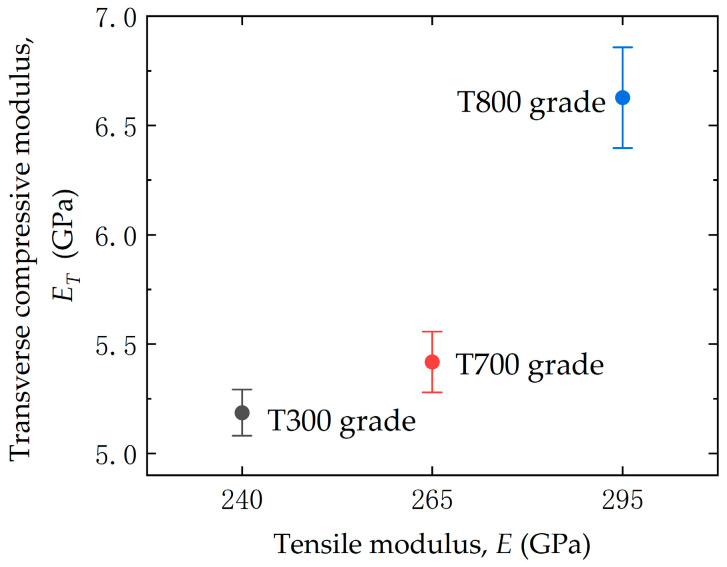
Relationship between transverse compressive modulus and tensile modulus for three domestic PAN-based single carbon fibers (T300, T700, and T800 grades).

Figure 8 shows the relationship between the transverse compressive modulus, $E_{T}$, and tensile modulus, E, of the three domestic high-strength PAN-based carbon fibers (T300, T700 and T800 grades). It can be clearly seen that there is a positive correlation between the compressive modulus and tensile modulus, the higher the tensile modulus, the higher the transverse compressive modulus for the PAN-based single carbon fibers.

The observed positive correlation between the transverse compressive modulus, $E_{T}$, and the tensile modulus, *E*, is due to the intrinsic microstructural anisotropy of PAN-based carbon fibers. This positive relationship aligns with theoretical expectations, as both moduli are governed by the alignment of graphitic crystallites and the density of structural defects. PAN-based carbon fibers exhibit a highly oriented graphitic layer structure along the fiber axis, which dominates tensile properties. However, transverse loading primarily engages weaker interlayer van der Waals bonds and shear deformation between crystallites [35]. Furthermore, this correlation is rooted in the degree of graphitic alignment and defect density. Higher-grade fibers (e.g., T800 grade) show larger crystallite sizes and improved orientation, enhancing both the transverse compressive modulus $E_{T}$ by reducing interlayer slippage and the tensile modulus *E* via simultaneously increased longitudinal stiffness [35,36]. Furthermore, these findings are consistent with the computational models presented by Zhang et al. [9], which attribute transverse stiffness to interlayer shear modulus and defect distribution.

### 3.2. Transverse Compressive Strength

The transverse compressive strength, $\sigma_{T}$, is defined as the breaking stress of carbon fiber and can be calculated according to Equation (6).

**Figure 9:**
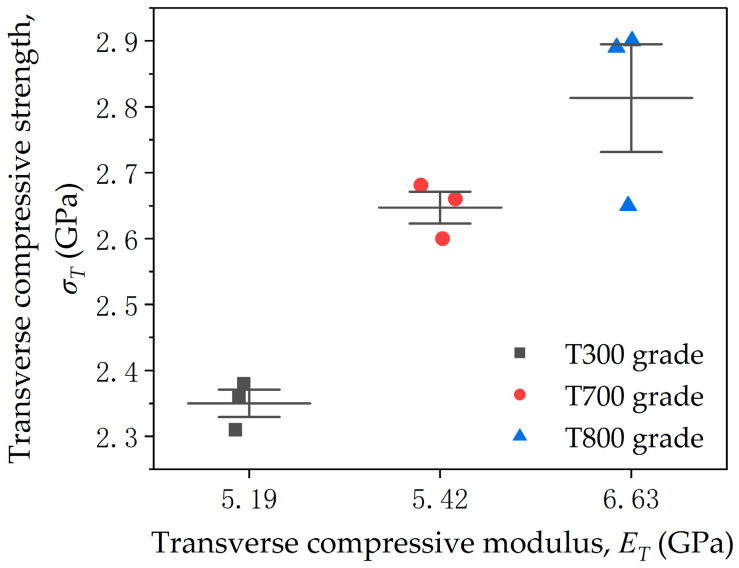
Relationship between transverse compressive strength and transverse compressive modulus for three domestic PAN-based single carbon fibers (T300, T700 and T800 grades).

In Figure 9, the transverse compressive strengths are plotted against the transverse compressive modulus. It is clear that the transverse compressive strengths increase with the compressive modulus.

**Figure 10:**
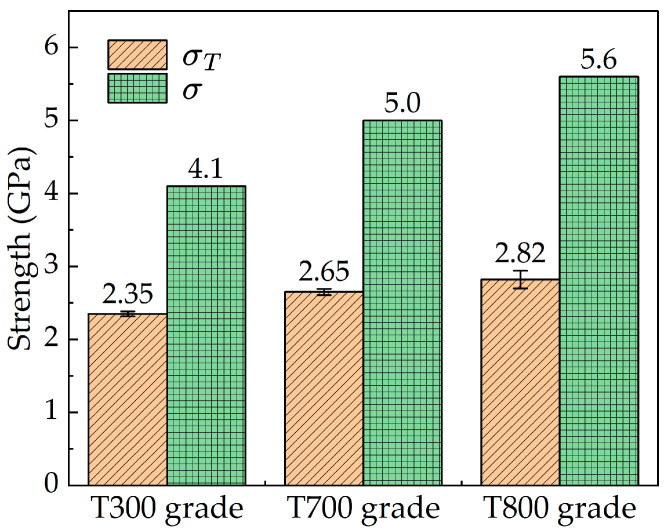
Relationship between transverse strength, $\sigma_{T}$, and tensile strength, σ, for three domestic PAN-based single carbon fibers (T300, T700 and T800 grades).

Table 2 compares the transverse compressive strength with tensile strength. The results are illustrated in Figure 10. The three domestic high-strength PAN-based single carbon fibers (T300, T700 and T800 grades) have compressive strengths of 2.35 GPa (standard deviation: 0.033), 2.65 GPa (standard deviation: 0.041), and 2.82 GPa (standard deviation: 0.121), respectively. The references tensile strength values are 4.1 GPa, 5.0 GPa and 5.6 GP. Reductions in strength of ~57.3%, 53.0% and 50.5% were observed. This result is coincident with the findings by Oya et al. [7,19], which is owing to the highly oriented anisotropic behavior of the carbon fibers. Notably, ellipticity causes stress concentration along the minor axis, reducing the measured $\sigma_{T}$ value due to initial failure initiation. The highest ellipticity (T300 grade: 1.25 ± 0.03) corresponds to the largest $\sigma_{T}$ underestimation (estimated at ~15% based on contact mechanics models [30]), while the T800 grade (ellipticity ratio of 1.09 ± 0.02) experiences only a ~5% reduction. Correcting for cross-section geometry would narrow the $\sigma_{T}$ gap between T300 and T800, though T800’s superior microstructure still dominates the trend.

Previous works have reported that CFRP composites with lower transverse compressive strength tend to develop cracks perpendicular to the fiber direction under impact loading [9,10,11]. These cracks can rapidly propagate along weaker interfaces (such as fiber–matrix interfaces or interlaminar regions), leading to delamination or catastrophic fracture, thus significantly compromising the energy absorption capacity of the CFRP laminates [16,17]. Owing to the significant anisotropy, transverse stress concentration tends to form localized high-stress zones at fiber-matrix interfaces or interlaminar regions under impact loading, accelerating the initiation and propagation of microcracks, which ultimately serve as initiation points for impact damage [19]. Moreover, studies have illustrated that lower transverse compressive resistance exacerbates interfacial debonding, particularly inducing delamination in CFRP laminates [10,37]. The detrimental effect of delamination on the impact resistance of composite materials has been quantified through Compression After Impact (CAI) tests [14,17].

### 3.3. Failure Mode

**Figure 11:**
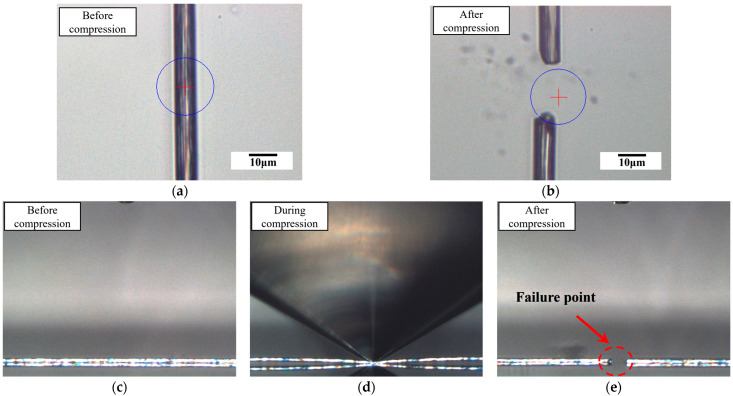
Typical optical macrographs for PAN-based single carbon fiber (T300 grade) before, during and after compression, (**a**,**b**) top view, (**c**–**e**) side view. Noted that the blue circle and red plus sign in (**a**) and (**b**) indicating the indenter and centering point respectively, as defined by the software.

Typical optical macrographs for PAN-based single-carbon fiber (T300 grade) before, during, and after compression are shown in Figure 11. The brittle failure mode can be observed in Figure 11b,e, which is coincident with the transverse compression curves (see Figure 6 and Figure 7a).

**Figure 12:**
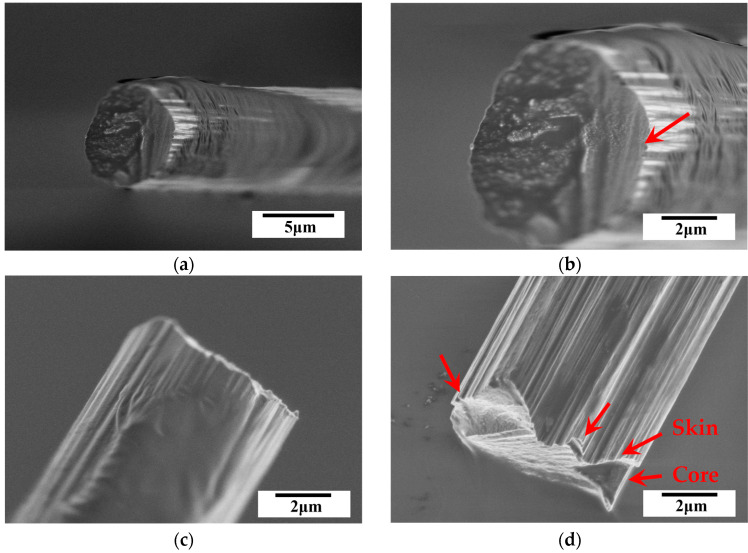
Typical SEM fractographs for PAN-based single carbon fiber (T300 grade) after compression, (**a**,**b**) top view, (**c**,**d**) side view. Noted that the arrows indicate the skin-core structure observed of single carbon fiber.

**Figure 13:**
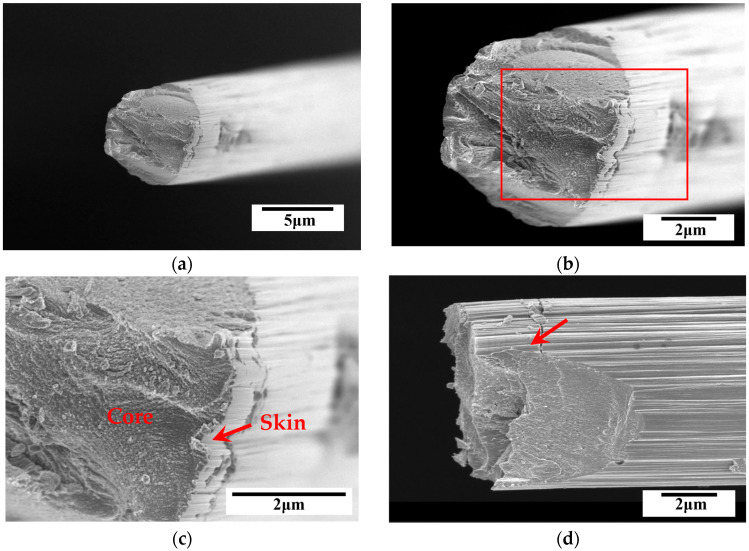
Typical SEM fractographs for PAN-based single carbon fiber (T700 grade) after compression, (**a**,**b**) top view, (**c**) higher magnification view of the framed area in (**b**), (**d**) side view. Noted that the arrows indicate the skin-core structure observed of single carbon fiber.

**Figure 14:**
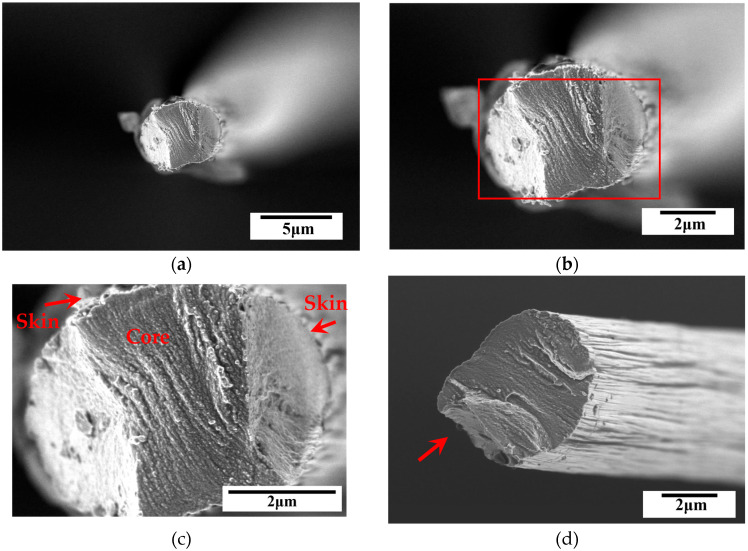
Typical SEM fractographs for PAN-based single carbon fiber (T800 grade) after compression, (**a**,**b**) top view, (**c**) higher magnification view of the framed area in (**b**), (**d**) side view. Noted that the arrows indicate the skin-core structure observed of single carbon fiber.

The fracture surface was analyzed from top and side views using SEM. The fractographs for the T300, T700 and T800 grades are shown in Figure 12, Figure 13, and Figure 14, respectively. A typical skin-core structure can be clearly seen in Figure 12b, where the arrow highlights localized collapse or wrinkling morphology indicating localized failure due to compressive forces. This observation indicates that the highly oriented graphite layer structure of the carbon fibers sustains interlayer sliding or buckling, leading to local structural instability under compression. Such morphology is directly associated with a low transverse compressive modulus [7,9]. Furthermore, the localized collapse or wrinkling serves as microscopic evidence of anisotropic mechanical behavior [19]. This anisotropy arises from the parallel alignment of graphite layers, where inefficient transverse load transfer across interlayers results in localized stress concentration. Consequently, the highly oriented graphite layer structure leads to a significantly lower transverse compressive modulus compared to the longitudinal modulus of single carbon fibers. Additionally, micro-defects (e.g., pores, impurities) introduced during fiber manufacturing may act as crack initiation sites for this localized collapse or wrinkling [9], severely degrading the compressive behavior of single carbon fibers.

Furthermore, SEM observations confirmed a brittle failure mode characterized by a relatively flat fracture surface. Despite all fibers exhibiting brittle failure, SEM analysis revealed subtle differences in fracture morphology across fiber grades. The T300 fibers displayed irregular fracture surfaces with localized delamination (indicated by arrows in Figure 12d), likely due to higher defect density and weaker interlayer bonding. In contrast, the T800 fibers showed smoother fracture planes (Figure 14b,d), indicative of reduced stress concentrations from defects and improved structural homogeneity. These variations align with statistical fracture mechanics, where flaw size distribution dictates failure patterns [36]. The superior graphitic alignment of T800’s likely suppressed crack growth, leading to more uniform failure. These observations indicate how microstructural refinement in higher-grade fibers mitigates defect-driven failure variability.

At higher magnifications, the skin-core structure observed via SEM (Figure 12d, Figure 13c and Figure 14c) directly impacts mechanical anisotropy. SEM analysis revealed quantifiable differences in skin-core thickness ratios (t_skin_/d_core_) across grades (0.12 ± 0.03 for T300, 0.18 ± 0.02 for T700, and 0.25 ± 0.04 for T800). Regression shows that $E_{T}$ scales linearly with skin-core thickness ratios (R^2^ = 0.92)—confirming that thicker, stiffer ‘skins’ in high-grade fibers reduce interlayer slippage (indicated by arrows in Figure 14c). This explains T800’s superior $E_{T}$ (6.63 GPa versus 5.19 GPa for T300 grade). In T300 fibers, core regions with disordered crystallites promote interlayer slippage (indicated by arrows in Figure 12b), reducing $E_{T}$. Higher grades of carbon fiber (e.g., T700 grade and T800 grade) with thinner cores (Figure 13b and Figure 14b) correlates with its higher $E_{T}$ values, this is consistent with composite lamination theory, where stiffer ‘skin’ layers dominate transverse load-bearing. Additionally, micro-defects (e.g., voids in T300 grade, see Figure 12d) act as stress concentrators, initiating cracks that propagate along weak interlayers. While defect quantification was not performed here, the positive relationship between $\sigma_{T}$ variability and fiber grades (Table 2) implies that higher-grade fibers benefit from stricter process controls (e.g., stabilization control [23]) and surface engineering (e.g., sizing chemistries [24]) to mitigate defects, a framework now accessible via integrated modeling tools [25].

Furthermore, as shown in Table 2, micro-defects (pores and impurities) significantly influence $\sigma_{T}$ scatter. The mechanical anisotropy ($E_{T}$ ≪ *E*) is well-described by composite lamination theory, where transverse properties are governed by matrix-dominated mechanisms. However, in single fibers, the ‘matrix’ is replaced by interlayer bonding. Advanced models, such as Mori–Tanaka homogenization [38], could extend this framework to account for skin-core heterogeneity and defect distributions. Statistical fracture models [39] further explain $\sigma_{T}$ variability, where weaker interfaces or larger defects act as critical flaws. These models align with our findings, emphasizing the need for microstructural optimization (e.g., reducing skin-core contrast) to enhance transverse performance in CFRP composites. While this work establishes fiber-level baselines, CAI testing of CFRP laminates using these fibers is planned to validate bulk impact performance. Preliminary simulations indicate that laminates with T800 fibers could achieve at least 10% higher CAI strength than T300-based equivalents, mitigating delamination through improved transverse load transfer [14,15].

## 4. Conclusions

In this study, the direct measurement of the transverse compressive properties of three domestic high-strength PAN-based single carbon fibers (T300, T700 and T800 grades) with diameters of ~5.5 μm–6.5 μm was made possible through the use of a commercialized micro-compression tester. This methodology establishes a standardized testing protocol that directly addresses limitations of prior approaches through integrated high-resolution displacement measurement (0.0001 μm), real-time optical alignment verification, and automated force-displacement control, significantly reducing machine compliance errors and manual intervention compared to custom-built systems. The tester provides not only the load–displacement curve during compression but also the specimen dimension measurements and real-time observation of compression behavior during testing. The relationships between the transverse compressive properties of the high-strength PAN-based single-carbon fibers were discussed. The fracture surface was also analyzed using both optical microscopy and SEM. The findings can be summarized as follows:

1. The transverse compressive moduli, $E_{T}$, for the T300, T700, and T800 grades are 5.19 GPa, 5.42 GPa and 6.63 GPa, respectively. For the three domestic PAN-based carbon fibers, the transverse compressive modulus increases with the increases in tensile modulus.
2. The transverse compressive strengths, $\sigma_{T}$, for the T300, T700, and T800 grades are 2.35 GPa, 2.65 GPa and 2.82 GPa, respectively. For the three domestic PAN-based carbon fibers, the transverse compressive strength increases with the increases in transverse compressive modulus, and it reduces to ~50% of tensile strength.
3. Two regions were found on the compression curves. In the initial incubation region, the fiber aligned between the indenter and sample stage, and the load increased slowly and non-linearly with displacement. In the second region, where the displacement increased linearly with load, and in this region the curve was well-fit by the Morris model.
4. The high-strength PAN-based carbon fibers show a brittle failure mode during transverse compression, coincident with the microstructure observations.
5. The quantified $E_{T}$ and $\sigma_{T}$ values provide critical inputs for multi-scale composite modeling, including the development of a meso-mechanical model for carbon fiber-reinforced polymer (CFRP) composites to investigate the influence of carbon fiber properties and carbon fiber-resin interface characteristics on the performance of composites in resisting impact damage. This framework thus explores the underlying mechanisms and establishes correlations between the impact damage resistance of laminated plates and fiber performance.

Based on the above findings, this study not only provides a basis for establishing standardized testing methods but also holds significant engineering value for improving carbon fiber production processes and the design and application of CFRP composites. While this study advances standardized testing, we acknowledge that non-ideal fiber geometries may lower the absolute modulus/strength values compared to idealized circular cross-sections. Future work should compare geometrically optimized fibers with conventional fibers to isolate microstructure effects. To extend this research, it is also worthwhile to conduct temperature-dependent testing, e.g., evaluating the transverse compressive modulus $E_{T}$ and strength $\sigma_{T}$ across temperatures ranging from −50 °C to 200 °C to assess performance in aerospace thermal cycles.

## Figures and Tables

**Table 1:** Basic information on single carbon fibers.

Fiber	Batch No.	Length, l (μm)	Diameter, D (μm)
T300 grade	300-1	50.01 (0.006)	6.41 (0.211)
	300-2	50.00 (0.015)	6.32 (0.339)
	300-3	50.01 (0.010)	6.49 (0.282)
	Average	50.01 (0.006)	6.41 (0.085)
T700 grade	700-1	50.01 (0.006)	6.10 (0.643)
	700-2	50.02 (0.021)	6.31 (0.811)
	700-3	50.01 (0.110)	6.25 (0.644)
	Average	50.01 (0.006)	6.22 (0.108)
T800 grade	800-1	50.00 (0.010)	5.13 (0.271)
	800-2	50.01 (0.006)	5.19 (0.347)
	800-3	50.01 (0.006)	6.14 (0.424)
	Average	50.01 (0.006)	5.49 (0.567)

() indicates standard deviations.

**Table 2:** Transverse compressive properties and tensile properties of three domestic PAN-based single carbon fibers (T300, T700 and T800 grades).

Fiber	Batch No.	Compressive Properties		Tensile Properties		Transverse-to-Tensile Modulus Ratio (%)	Transverse-to-Tensile Strength Ratio (%)	Failure Mode
		$\mathrm{Modulus},\;E_{T}$ (GPa)	$\mathrm{Strength},\;\sigma_{T}$ (GPa)	Modulus, E (GPa)	Strength, σ (GPa)			
T300 grade	300-1	5.15 (0.183)	2.31 (0.273)	240	4.1	2.2	57.3	Brittle
	300-2	5.24 (0.075)	2.38 (0.290)					
	300-3	5.17 (0.172)	2.36 (0.247)					
	Average	5.19 (0.050)	2.35 (0.033)					
T700 grade	700-1	5.45 (0.255)	2.66 (0.147)	265	5.0	2.1	53.0	Brittle
	700-2	5.51 (0.120)	2.68 (0283)					
	700-3	5.30 (0.137)	2.60 (0.149)					
	Average	5.42 (0.104)	2.65 (0.041)					
T800 grade	800-1	6.49 (0.314)	2.68 (0.446)	295	5.6	2.2	50.5	Brittle
	800-2	6.67 (0.275)	2.89 (0.126)					
	800-3	6.72 (0.353)	2.90 (0.115)					
	Average	6.63 (0.120)	2.82 (0.121)					

() indicates standard deviations.

## Data Availability

The original contributions presented in this study are included in the article. Further inquiries can be directed to the corresponding authors.
